# Supplementary material for: Patterns of Intron Gain and Loss in Fungi
Source: PLoS Biol. 2004 Nov 30;2(12):e422. doi: 10.1371/journal.pbio.0020422 (PMC532390; doi:10.1371/journal.pbio.0020422)
Supplement: Table S1 — Also available at http://genes.mit.edu/NielsenEtAl/. (4.3 MB ZIP). [file pbio.0020422.st001.zip › NielsenEtAl/html/1307.html]

AN3084.1.NCU06655.1.MG06006.1.FG10308.1


```
 CLUSTAL W (1.82) Multiple Sequence Alignments - Introns Inserted


Sequence 1: NCU06655.1	436 aa
Sequence 2: MG06006.1	445 aa
Sequence 3: FG10308.1	444 aa
Sequence 4: AN3084.1	465 aa
Alignment Length: 469 aa
Number Identitical Residues: 142 aa
Alignment Score (without introns) 8560


MG06006.1 	---METVLASSGGTARADHLCVLVHG0LWGNPAHMAQVAKALRDRYSRDQLYIIVAKRNS
NCU06655.1	------MADYIGGNIEADHLCVLVHG0LWGNPNHMASVAKALRAQYPRDKLNILVAKRNA
FG10308.1 	MSKPVVLEDVTGGSVNATHLCVLVHG0LWGNPSHLRNVAKSLRDNYSEDELYILLAKQNS
AN3084.1  	---MDPDSDNWTTAPKADHLCVLVHG2LWGNPSHMGSIASALRKRYDEKQLHILVAECNT
          	               .* ******** ***** *: .:*.:** .* ..:* *::*: *:

MG06006.1 	GSFTYDGIELGGQRVCREIEEELEKIKKTGGKITKISFIGYSMGGLVARYAIGLLEAKGV
NCU06655.1	GSFTYDGIELGGERVCNEIEEELQAVESKGGKIKKISIAGYSLGGLVARYAIGLLYARGV
FG10308.1 	GNHTYDGIETGGERVCAEIEQELKDIEKKGGKITKLSIAGYSLGGLVSRYAVGLLYAKGV
AN3084.1  	GNLTYDGIEVCGERLAHEIEATLSKLEADGHKMRKLSVVGYSLGGLISRYAIGLLYARGW
          	*. ******  *:*:. ***  *. ::  * *: *:*. ***:***::***:*** *:* 

MG06006.1 	LEK--LQCI0NFTAFASPFLGCRTPLKGWNNHLFNVLGARTLSLSGRQLFGIDKFRDTGR
NCU06655.1	LDN--LECM0TFTAFASPFLGVRTPLRGWPNHVWNVLGARTLCMSGRQLFGIDHFRDTGK
FG10308.1 	LDD--LECM0NFTTFATPHLGVRTPLKGWLSNIYNVLGARTLSMSGRQLFTIDSFRNTNR
AN3084.1  	LDDDKLEPV~NFTTFASPHLGARAPVRGVQNLLFNGLGSRTISTSGKQMFLADTFQDTGK
          	*:...*: : .**:**:*.** *:*::*  . ::* **:**:. **:*:*  * *::*.:

MG06006.1 	PLIAVMTDQESIFMSGLRRFKRHTLYSNIVNDRAAVYYTTSISKTDPFVDLDKVKLNFVE
NCU06655.1	PLLAVLADPKSIFMCGLAKFKRRILYTNIVNDRSAVYYTTGIAKTDPYTDLDKVKVNYVK
FG10308.1 	PLLAVLADPNSIFMSGLKKFKRRTLYANIVNDRSVVHYTSGINKTDPYTDLDNVKLNYLK
AN3084.1  	PLLSALADPNSIFIEGLKRFKNRCVYGNVVNDRTTAFYTTIFASEDHFRDLENKTIKYVK
          	**::.::* :***: ** :**.: :* *:****:...**: : . * : **:: .:::::

MG06006.1 	GYENVILDPDCPVR-----PKERVAP-----TAFKALKNAPFFLAMAVLLPIGITAFLLN
NCU06655.1	GYEPVILDPTNPVSPAPPKPKEPLVA-----NLKKWINRVPFILAVAVFVPLGLVAFFIN
FG10308.1 	DYDGVILDPDHPVTQSPKVQGQDTLLS-DFDSIKKWIKNVPFMLTIGVIIPIGVVVFLAN
AN3084.1  	GYEPVVIDPDVYTLPASEDKKRPPFPLRAWQSIRTFFSKLPFWLFFCLILSIAIPVFLVY
          	.*: *::**   .  :.    .        ..  . :.. ** * . :::.:.: .*:  

MG06006.1 	AGVQTARSSKRLRAHRQSHDSKNYS--KLPLWVKEVRGALEDAYENLNSSQRQEFLAS--
NCU06655.1	SAVQTVRSSKRVKLHEKGLAGIDVESYRVDLWIKEMREAVEHAYEDLNSSQNQEYLGL--
FG10308.1 	SAVQTVRSAKRIKLHESGLAGLKIEDYRMPLRIKAIQEEVEQAYESFNSAQDQQYLAS--
AN3084.1  	SVRETFRSRERIREHEEGP-GKSFRSYRMPLGLRRIQTAMDEVFENAAARQDPDYISPNV
          	:  :* ** :*:: *...  . .  . :: * :: ::  ::..:*.  : *  :::. . 

MG06006.1 	-----ETPSEDEGAGGAADDRASTMGVEKAEDDSRLALERKQSHAAMPTLALAPYQFSMI
NCU06655.1	-----SDGDLDEET----DD---------SSNQEILALERKQSHPHQPTLALAPYQFAAI
FG10308.1 	-----DSDDDLAYD---------------AEDRQKLRRERRMSTVEQPTLALTPDQFEMI
AN3084.1  	PTTDLESQTASRNDKTGTAEDSPAAASSGNSTDDANKVKKVRCGEKRPTIALTPDQFSMI
          	.::. .          .: . :.: . .  .  .    ::  .    **:**:* **  *

MG06006.1 	DSLDSLGWRKYRVWIQKNNHSHAAIIVRIVRDSYSEGYQVLKHWLDNEFII
NCU06655.1	NALDALGWRKYPVWIHNDRHSHAAIIVRMEKPTFEEGKVVLSHWVNEEFLI
FG10308.1 	ENLNAAGWRKFPVHIQKHRHSHAAIVVRMDKESFADGWVVLGHWAGSEFLI
AN3084.1  	KSLNAVGFRKYPVYIHNHEHTHAAIVVRMDDPGYAEGHTVIKHWLDQEFDL
          	. *:: *:**: * *::..*:****:**:    : :*  *: ** ..** :
```
